# Research Progress on the Mechanism of Natural Product Ingredients in the Treatment of Uveitis

**DOI:** 10.1155/2021/6683411

**Published:** 2021-07-16

**Authors:** Sicong Li, Fang Liu, Kai Zhang, Yujia Tong, Xin Liu

**Affiliations:** ^1^School of Pharmacy, Peking University Health Science Centre, Beijing, China; ^2^Department of Pharmacy, China-Japan Friendship Hospital, Beijing, China; ^3^Department of Rheumatology and Immunology, Peking University People's Hospital, Beijing, China; ^4^Institute of Medical Information, Chinese Academy of Medical Sciences/Peking Union Medical College, Beijing, China; ^5^School of Traditional Chinese Medicine, Beijing University of Traditional Chinese Medicine, Beijing 100029, China

## Abstract

**Background:**

As the spectrum of ophthalmic diseases keeps changing, uveitis has gradually become one of the major blinding eye diseases in the world. In recent years, it has become a research hotspot to select effective components for uveitis treatment from natural drugs.

**Methods:**

We searched PubMed and EMBASE databases for studies written in English as well as Chinese National Knowledge Infrastructure (CNKI), CQVIP, and Wan Fang database for studies written in Chinese (inception through 30 December 2020).

**Results:**

Eight kinds of natural product ingredients were included in this article. They were found to not only regulate the expression of cytokines, proliferation, and differentiation of T help cells but also inhibit the damage of cytokines and inflammatory cells to uvea, blood aqueous barrier, and blood retinal barrier.

**Conclusion:**

Natural product ingredients have their unique advantages in the treatment of uveitis. They have good anti-inflammatory effects without causing serious adverse reactions, which enables them to be promising choices for preventive and therapeutic strategy of uveitis.

## 1. Introduction

Uveitis refers to various intraocular inflammatory diseases occurred in uvea (i.e., iris, ciliary body, and choroid) and its adjacent structures (including cornea, vitreous body, retina, and optic nerve) [1]. Without timely diagnosis and treatment on chronic inflammation in the eye, it will lead to cataracts, glaucoma, corneal lesion, macular edema, or even permanent vision loss [2]. Uveitis can be divided into three categories according to its pathogenesis: infectious uveitis caused by pathogens like bacteria, viruses, and fungi and autoimmune-related uveitis as well as camouflage syndrome. Rheumatoid arthritis, Behcet's disease, and inflammatory bowel disease as well as juvenile idiopathic arthritis are often accompanied by uveitis [3].

**Figure 1:**
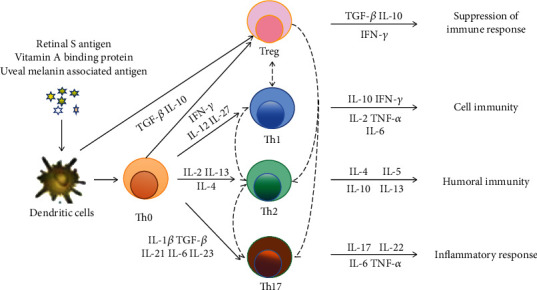
T lymphocytes and related immune responses in uveitis.

The abnormal number and function of cluster of differentiation 4 + (CD4 +) T cells play important roles in the immunopathogenesis of uveitis [4]. As is shown in Figure 1, retinal S antigen, vitamin A binding protein between photoreceptors, and uveal melanin-associated antigen excessively activate dendritic cells that promote the differentiation of CD4 + T cells into different subtypes, such as T helper 1 cells (Th1 cells), T helper 2 cells (Th2 cells), regulatory T cells (Tregs), and T helper 17 cells (Th17 cells) [5].

Interferon-*γ* (IFN-*γ*), interleukin-12 (IL-12), and interleukin-27 (IL-27) induce differentiation of Th1 cells that secrete cytokines, including interleukin-2 (IL-2), IFN-*γ*, and tumor necrosis factor (TNF-*α*) and participate in cellular immunity [6]. The expression levels of TNF-*α* and IFN-*γ* are positively correlated with the severity of uveitis [7]. IL-1*β*, transforming growth factor-*β* (TGF-*β*), interleukin-12 (IL-12), interleukin-6 (IL-6), and interleukin-23 (IL-23) induce differentiation of Th17 cells that mediate the immune response. Both Th1 and Th17 cells play important roles in the pathogenesis and recurrence of uveitis [8]. IL-2, IL-4, and IL-13 induce differentiation of Th2 cells that participate in humoral immunity [9]. Functions of Th-2 can be inhibited by IFN-*γ* that secreted by Th1 cells [10]. TGF-*β* and IL-10 can induce the differentiation of regulatory T cells (Treg cells) that inhibit the function of Th1 and Th17 cells by secreting TGF-*β* and IL-10 [11]. Less and impaired functions of Tregs are observed in uveitis patients [12]. It can be concluded that regulating immune function is an effective way in the treatment of uveitis.

Most of the current research on uveitis drugs focuses on biological agents. Infliximab, a tumor necrosis factor antagonist, can effectively treat vitreous opacity, active retinal vasculitis, and macular cystic edema caused by uveitis and scleral inflammation [13, 14], but it can lead to tuberculosis and aggravate demyelination disease. Adalimumab has been proved to be effective and safe for uveitis treatment in many trials [15–18] but has not been approved by the National Medical Products Administration (NMPA) for clinical treatment of uveitis in China. The main drugs currently available in China to treat uveitis include immunosuppressants and glucocorticoids. Immunosuppressants such as cyclophosphamide, cyclosporin, and azathioprine not only inhibit bone marrow function but also have nephrotoxicity or hepatotoxicity [19]. Subconjunctival injection, peribulbar injection, retrobulbar injection, and vitreous cavity injection of glucocorticoid can improve visual impairment, inhibit the formation of adhesion, and relieve eye pain but can lead to adverse reactions including ptosis, cataract, and increased intraocular pressure [20]. Oral administration of corticosteroids can treat uveitis by inhibiting the destruction caused by the inflammatory response. Expansion of capillaries and proliferation of fibroblasts can be inhibited by corticosteroids [21]. On the other hand, corticosteroids can lead to peptic ulcers, hypertension, and hyperlipidemia [22]. In recent years, it has become a hot spot in the research field of uveitis to extract immunomodulators with high efficiency and low toxicity from natural products. In this article, the therapeutic effects of alkaloids, glycosides, polysaccharides, and polyphenols on uveitis and their mechanisms were discussed in detail, to provide a reference for drug development and clinical research.

## 2. Materials and Methods

### 2.1. Search Strategy

PubMed and EMBASE databases were searched for studies written in English. Chinese National Knowledge Infrastructure (CNKI), CQVIP, and Wan Fang databases were searched for studies written in Chinese. We searched for articles published before 30 December 2020. Keywords including “Uveitis” or “Panuveitis” plus any of the following: “natural product”, “Matrine”, “Berberine”, “Total glucosides of paeony”, “Tripterygium wilfordii polyglycoside”, “Astragalus polysaccharide”, “Hedysari polysaccharide”, “Rhubarb polysaccharide”, and “Curcumin” were used to search for articles in Chinese database. The search strategy for articles written in English is shown in Tables 1 and 2.

### 2.2. Study Inclusion and Exclusion Criteria

#### 2.2.1. Inclusion Criteria

The inclusion criteria are the following: (1) articles about studies of natural product ingredients' therapeutic effect on uveitis, (2) written in Chinese or English, and (3) abstract and full text available.

#### 2.2.2. Exclusion Criteria

**Figure 2:**
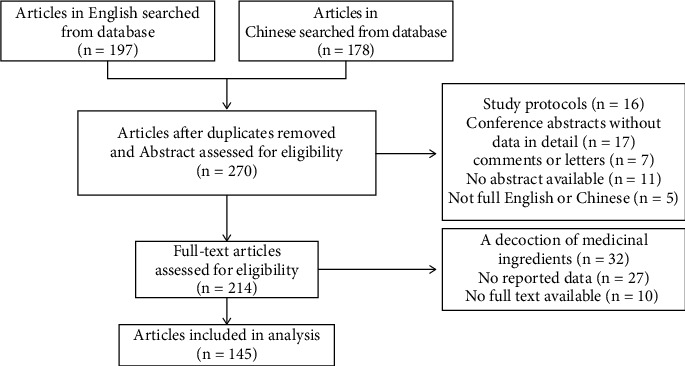
Flow chart of literature screening.

The exclusion criteria are the following: (1) study protocols, conference abstracts without data in detail, comments, or letters; (2) no data reported; and (3) therapeutic drug is a decoction of medicinal ingredients. Process of literature screening is revealed in Figure 2.

## 3. Results and Discussion

### 3.1. Alkaloids

Alkaloids are nitrogen-containing alkaline compounds widely found in natural products. Among them, matrine and berberine have anti-inflammatory effects.

#### 3.1.1. Matrine

Extracted from the dried root of *Sophora flavescens Alt.*, matrine has antibacterial, anti-inflammatory, antitumor, and antiarrhythmia effects. Matrine has an inhibitory effect on the inflammatory reactions caused by liposaccharides (LPS) [23, 24] LPS, a component of the cell wall of gram-negative bacteria, acts on the Toll-like receptors 4 (TLR4) receptor to induce myeloid differentiation factor88 (MyD88) recruitment, followed by activation of nuclear factor kappa-B (NF-*κ*B) through a series of phosphorylation cascades [25]. After the inflammatory factors destroy the blood-aqueous barrier or blood-retinal barrier, some macromolecular protein substances and cells in the blood infiltrate into the interstitial or intracavity of the eye (anterior chamber or vitreous body), giving rise to different degrees of tissue damage [26]. The breakdown of the blood-aqueous barrier leads to iris neovascularization. The wall of the neovascularization is susceptible to rupture and leads to hyphema [27]. Matrine eye drops (low dosage group (0.50 g/L), middle dosage group (0.75 g/L), high dosage group (1.00 g/L)) decreased the IL-6, IL-1, and TNF-*α* level in serum and aqueous humor of the uveitis model rabbits induced by LPS; inhibited the expression of the TLR4, MyD88, and NF-*κ*B p65 in retinal tissue; and improved rabbit ciliary hyperemia, retinal edema, and retinal fundus bleeding [28]. Subconjunctival injection of matrine (0.8 mg) inhibited the expression of vascular endothelial growth factor (VEGF) mRNA in the corneal tissue burnt by alkali [29].

If inflammatory cells and mucin deposits in aqueous humor block trabecular meshwork and impede the outflow of aqueous humor, it will increase intraocular pressure and give rise to glaucoma in uveitis patients. Trabeculectomy is a commonly used treatment. After trabeculectomy, putting 1.0 g/L matrine cotton tablets under the scleral flap for 28 days reduced the proliferation of fibroblasts and reduce the formation of filter bubble scar [30].

#### 3.1.2. Berberine (BBR)

Extracted from *Coptis chinensis Franch.*, berberine is a kind of isoquinoline alkaloid with anti-inflammatory, antitumor, antibacterial, and antiviral effects. The therapeutic effect of berberine on uveitis has been confirmed in animal and in vitro experiments.

T helper cell 17 (Th17) cells play an important role in the pathogenesis of ocular Behcet's disease and uveitis [31]. IL-6, IL-21, IL-23,TGF-*β*, or IL-1*β* drive the production of IL-17 in Th17 cells by activating signal transducer and activator of transcription-3 (STAT-3) and retinoid-related orphan nuclear receptor *γ*t (ROR*γ*t) [32]. In vitro, berberine (5 *μ*M) not only inhibited Th17 and Th1 cell differentiation and secretion of IL-17 and IFN-*γ* but also regulated the balance of T regulatory cell (Treg)/T helper cell (Th17) in patients with ocular Behcet's disease [33]. Cytokines such as IL-6, IL-1, and IL-23 secreted by dendritic cells (DC cells) drive the differentiation and production of interleukin-17 by activating STAT-3 [33, 34]. BBR downregulated the expression of costimulatory molecules, including clusters of differentiation 40 (CD40), clusters of differentiation 80 (CD80), and clusters of differentiation 86 (CD86) and inhibited DC cells' maturation and the secretion of IL-6, IL-1, and IL-23 [35]. Uveitis may be accompanied by retinal pigment epithelium lesions, retinal edema, thinning, and hemorrhage. Berberine dose-dependently inhibited dysfunction of the blood-retina barrier induced by IL-1 and improved retinal edema in rats [36].

In vivo, berberine's therapeutic effect on uveitis has also been proved. Interleukin-8 and monocyte chemotactic protein-1 (MCP-1) play important roles in LPS-induced uveitis. Cytokine-induced neutrophil chemokine-1 (CINC-1) is a rat analog of IL-8. The role of IL-8 and CINC-1 in endotoxin-induced uveitis has been confirmed by many studies [37, 38]. Orally taking a 0.8 mL berberine solution before injecting LPS in Wistar rats inhibited the expression of MCP-1 mRNA and CINC-1 mRNA of the iris ciliary and the damage of the iris ciliary body caused by inflammatory cells [39]. Li et al. [40] intraperitoneally injected berberine (2 mg/kg) into experimental autoimmune uveitis (EAU) rats for two weeks and found only a slight inflammatory reaction in the eyes, while in the normal saline group, serious vasodilation, iris hemorrhage, and purulent exudation were observed. The therapeutic effect of berberine on experimental autoimmune retinitis is also related to regulating intestinal flora. After 14 days of intragastrically administrating berberine (100 mg/kg), the number of Th1 and Th17 cells in the spleen of mice significantly decreased, while the number of Treg cells increased. Berberine inhibited the breakdown of the blood-retinal barrier, and corneal edema, retinal folding, iris congestion, and iris adhesion were significantly improved, which was related to the significant changes in the composition of the spleen transcriptome and intestinal microorganisms. The dominant microbiome in the experimental autoimmune uveitis (EAU) group was Lactobacillus aceae, while in the BBR group was Muribaculaceae. By means of MetaStat analysis, five genera, including Lactobacillus, was reduced, with thirteen genera, including Akkermansia and Oscillibacter, increased in the BBR group. Compared with the EAU group, 249 differentially expressed genes (DEGs) were downregulated, and 227 DEGs were upregulated. The downregulated biological processes mainly included nucleosome assembly, chromatin assembly, myelin differentiation, and antigen processing and expression. It suggested that berberine led to significant changes in the overall transcription profile of genes [41].

### 3.2. Glycosides

Glycosides are compounds formed by the attachment of end-group carbon atoms of a sugar or sugar derivative to another type of nonsugar substances (called glycosides, ligands, or glycosides). Most of the glycosides are colorless and soluble in water. With good anti-inflammatory activity, total glucosides of paeony (TGP) and Tripterygium wilfordii polyglycoside (TWP) are widely used in the clinical treatment of rheumatic immune diseases in China.

#### 3.2.1. Total Glucosides of Paeony (TGP)

Extracted from the dried root of *Paeonia lactiflora Pall.*, TGP have anti-inflammatory, analgesic, immunomodulatory, and antitumor effects. TGP capsule has been approved for clinical treatment of rheumatoid arthritis in China since 2005. Moreover, TGP have been reported to be used in the clinical treatment of rheumatoid arthritis [42], idiopathic arthritis [43], systemic lupus erythematosus [44], and Sjogren's syndrome [45] in China.

The occurrence of uveitis is closely related to the dysfunction of T lymphocytes, especially autoreactive T lymphocytes. Activation-induced cell death pathway (AICD) plays a crucial role in maintaining immune tolerance and clearance of autoreactive T lymphocytes [46, 47]. Fas/FasL can induce faster apoptosis of T lymphocytes. TGP enhanced Bcl-2 expression in the EAU group's retinal tissues, which was very weak in the normal retinal tissues [48]. In cell experiments, total glucosides of paeony significantly inhibited T lymphocyte proliferation and promoted activation-induced T lymphocyte death by upregulating Fas and downregulating Bcl-2 expression [49].

In animal experiments, total glucosides of paeony (4.8 g/kg, once every 6 h, three times in total) were administered to rats with uveitis before LPS injection, which not only inhibited the invasion of inflammatory cells into the anterior chamber and vitreous body but also inhibited the swelling of iris and ciliary body and thickening of retinal edema, as well as fibrinoid exudation in the anterior chamber. Besides, it significantly alleviated iris bleeding, anterior chamber pus, and pupil narrowing [50]. Total glucosides of paeony (orally taken for 12 days) regulated the expression levels of IL-4 and IFN-*γ* genes in experimental autoimmune uveitis (EAU) rats and increased the expression levels of natural killer T cells [51].

In clinical trials, Xu et al. [52] treated 38 patients who suffered from systemic lupus erythematosus associated with uveitis, with compound tropicamide eye drops (four times a day) and local administration of tobramycin eye drops (four times a day). In addition to the medicines above, total glucosides of paeony (0.6 g, three times a day) were administered orally to 40 patients in the treatment group. Two months later, the total effective rate of patients in the treatment group was significantly better than that in the control group (95.00% vs. 78.95%, *P* < 0.05); the first withdrawal time of glucocorticoids in the treatment group was earlier than that in the control group (6.88 ± 1.721 days vs. 8.22 ± 1.98 days, *P* < 0.05).

#### 3.2.2. Tripterygium wilfordii Polyglycoside (TWP)

Extracted from the dried roots of *Tripterygium wilfordii Hook. F.*, Tripterygium wilfordii polyglycoside (TWP) has anti-inflammatory, antitumor, and immunomodulatory effects. TWP tablets have been approved in the treatment of nephrotic syndrome, Behcet's disease, and autoimmune hepatitis in China. Moreover, TWP has been used in the clinical treatment of immune diseases such as rheumatoid arthritis [53], systemic lupus erythematosus [54], and lupus nephritis [55] in China.

TWP can act on the TLR-NF-*κ*B signaling pathway in vitro [56]. TWP (15.27 *μ*mol/L) downregulated the expression of TLR4 and NF-*κ*Bp65, inhibited the endotoxin-induced inflammatory response in macrophages, and suppressed the release of TNF-*α*, IL-1*β*, IFN-*γ*, intercellular adhesion molecule 1 (ICAM-1), and monocyte chemotactic protein 1 (MCP-1), with effects superior to those of 0.19 *μ* mol/L of dexamethasone and 6.62 *μ*mol/L of azathioprine [57]. Matrix metalloproteinase 9 (MMP-9) can regulate the activity of cytokines like IL-8 and promote the release of vascular endothelial growth factors to participate in angiogenesis. Increased expression of MMP-9 is associated with experimental autoimmune uveitis [58] and endotoxin-induced uveitis [59]. Tripterygium wilfordii polyglycosides can dose-dependently inhibit the expression of MMP-9 and proinflammatory cytokine IL-32 [60]. IL-37 significantly inhibits the production of IL-1*β*, IL-6, IL-10, IL-21, IL-23, TNF-*γ*, and IFN-*γ* [61]. Tripterygium wilfordii polyglycosides (15 *μ*g/mL) also upregulated the expression of anti-inflammatory cytokine IL-37 through extracellular regulated protein kinases1/2 (ERK1/2) and p38 mitogen-activated protein kinase (MAPK) signaling pathways [62].

In clinical studies, Huang et al. [63] conducted a randomized controlled trial that proved the efficacy of Tripterygium wilfordii polyglycosides in the treatment of acute uveitis. The basic treatment was 1% atropine eye drops +0.05% dexamethasone eye drops. On this basis, 50 patients in the treatment group orally took Tripterygium wilfordii polyglycoside tablets (TWP) (20 mg, bid, for 4 weeks), while 50 patients in the control group orally took dimorpholine (0.4 g, TID, for 4 weeks). The effective rate of the two groups was 95.7% vs. 95.8%, without statistically significant difference. Ma [64] gave 1% atropine eye drops (three times a day) to 22 patients with recurrent uveitis combined with oral Tripterygium wilfordii polyglycoside tablets (20 mg, three times a day). The clinical effective rate was 95.6% after one week of treatment.

Approximately 50-87% of patients with Behcet's disease initially present with uveitis in one eye. Among them, anterior uveitis is the most common type [65]. 30 patients with ocular Behcet's disease orally took Tripterygium wilfordii polyglycoside tablets (30 mg/d) for 3 months, the serum levels of IL-1*β*, TNF-*α*, and IFN-*γ* significantly decreased, and the clinical effective rate was 86.6% [66]. Yang et al. [67] found that oral administration of Tripterygium wilfordii polyglycoside tablets (20 mg, bid, 2 months) could inhibit the expression of nitric oxide, soluble intercellular adhesion molecule (sICAM-1), and soluble vascular cell adhesion molecule (sVCAM-1) in plasma of 30 patients with ocular Behcet's disease and improve endothelial dysfunction.

### 3.3. Polysaccharide

With the characteristics of biodegradability, little toxicity, and side effects, the polysaccharide is a kind of important biological macromolecule composed of a variety of same or different monosaccharides with *α*- or *β*-glycosidery bonds [68].

#### 3.3.1. Astragalus Polysaccharide (APS)

Extracted from the root of *Astragalus mongolica*, Astragalus polysaccharide has pharmacological effects such as immune regulation, anti-inflammatory, antibacterial, antioxidant, improvement of microcirculation, and antitumor effects.

In cell experiments, APS inhibited LPS-induced inflammatory response by inhibiting the TLR4/NF-*κ*B pathway [69]. APS dosage-dependently inhibited the activation of NF-*κ*B and phosphorylation of ERK and c-Jun N-terminal kinase (JNK) to inhibit the production of TNF-*α* and IL-1*β* in LPS-stimulated macrophages [70]. APS (1.0 mg/mL) inhibited LPS-induced inflammatory response in mouse retinal ganglion cells by inhibiting tumor necrosis factor-associated receptor factor 6 (TRAF6)/transforming growth factor-*β* activated kinase 1 (TAK1) pathway [71].

Caspase-3 is a key effector in apoptosis. Caspase-3 also induces apoptosis in the nucleus, resulting in fragmentation of DNA and chromatin consolidation. In vitro experiments, by inhibiting the production of apoptotic factor caspase-3, 250 *μ*g/mL APS inhibited the necrosis of human retinal pigment epithelial cells caused by 100 *μ*mol/L hydrogen peroxide [72, 73]. Uveitis can be complicated by glaucoma. Injecting 1 mL methylcellulose after extracting 0.1 mL aqueous humor could produce acute high intraocular pressure model rats. In this model, APS (500 mg/kg) intragastric administrated for 14 days lowered intraocular pressure and relieved retinal edema. Moreover, APS inhibited retinal caspase-3 expressions to reduce retinal ganglion cell apoptosis. The thickness of the whole retinal layer, optic fiber layer, and the outer granular layer of the Astragalus polysaccharide group was significantly larger than that of the uveitis model group [74].

#### 3.3.2. Hedysari Polysaccharide (HPS)

Extracted from the dry root of *Hedysarum polybotrys Hand.-Mazz.*, Hedysari polysaccharide has antitumor, antioxidation, anti-inflammation, and antivirus effects [75]. Hedysari polysaccharide (400 mg/kg) reduced the clinical severity of endotoxin-induced uveitis in rats and inhibited the fibrin exudation and inflammatory cell infiltration in the eyes.

Toll-like receptor 4 (TLR-4) is the primary signal cell receptor recognized and activated by lipopolysaccharide. TLR-4 plays a key role in the onset of uveitis and eventually leads to the activation of inflammatory cytokines and inflammation pathological reactions [76]. Li et al. [77] found that the TLR4 signaling pathway involved in the pathogenesis of acute anterior uveitis. After bound to LPS, TLR4 produced proinflammatory cytokines that upregulated costimulators and major histocompatibility complex (MHC). After that, dendritic cells got activated, and their antigen presentation capacity got enhanced, followed by the initial T cells activated [78]. Activation of the NF-*κ*B signaling pathway was closely associated with inflammatory factor expression and extracellular matrix metabolic imbalance [79]. The TLR4/NF-*κ*B signaling pathway was an important pathway for regulating TNF-*α* and IL-1*β* expression. Activation of the TLR4-MD2-CD14 complex led to phosphorylation of the NF-*κ*B p65 subunit through a cascade of MyD88-dependent pathways [80], which allowed NF-*κ*B to be colonized in the nucleus and activate the expression of a variety of inflammatory mediators, including TNF-*α* and IL-1*β*. HPS significantly reduced the mRNA and protein expressions of TLR4, MyD88, tumor necrosis factor receptor-associated factor 6 (TRAF6), and NF-*κ*B65 [75, 81]. The glycogen synthase kinase 3-*β* (GSK3-*β*) in the TLR4 signaling pathway plays an important role in maintaining the immune system's balance. Yang et al. [82] found that intraperitoneally injecting HPS (400 mg/kg) into rats with uveitis induced by endotoxin upregulated the phosphorylation level of GSK3-*β* protein and inhibited the expression of nuclear factor-*κ*B (NF-*κ*B) P65 mRNA. As a result, the level of the anti-inflammatory factor IL-10 in the anterior chamber water was upregulated, while inflammatory cytokines such as TNF-*β*, IL-6, and lL-1*β* were inhibited, thereby inhibiting the damage of the uveium caused by the inflammatory response.

#### 3.3.3. Rhubarb Polysaccharide (RP)

Extracted from the dried roots and rhizomes of *Rheum palmatum L*., *Rheum officinale Baill*., and *Rheum tanguticum Maxim. ex Balf.*, Rhubarb polysaccharide has anti-infection, anti-inflammation, immune regulation, hypoglycemia, and antitumor effects. Rhubarb polysaccharides inhibited CD4 T cell proliferation and regulated cytokines produced by Th1 and Th2. Human leukocyte antigen-B27- (HLA-B27-) associated acute preuveitis is a common kind of uveitis, accounting for 18% to 32% of all cases. It is an acute inflammatory exudative disease of the iris ciliary body, with an urgent onset and rapid progression. If not effectively treated in time, patients can develop into severe intraocular complications, such as glaucoma, which eventually leads to blindness. After activated by LPS, TLR4 activated NF-*κ*B through the MyD88-dependent pathway to promote the release of cytokines such as NO and TNF-*α*, thus initiating the damage of immune cells towards the uveal membrane. In vitro, rhubarb polysaccharide (100 mg/L) had a protective effect on monocytes of rats with HLA-B27-associated acute preuveitis [83]. Rhubarb polysaccharide inhibited the TLR4/NF-*κ*B signaling pathway and inhibited the secretion of TNF-*α*, IL-10, IL-17, INF-*γ*, and IL-1*β*, with no significant difference from the therapeutic effect of monoclonal antibody against TLR4 (5 mg/L) [25].

### 3.4. Polyphenol

Having phenolic structures with multiple hydroxyls, polyphenols are the secondary metabolites of plants that widely existed in fruits, vegetables, and herbal medicines. Among them, curcumin has an anti-inflammatory effect.

#### 3.4.1. Curcumin

Extracted from the tuberous root of *Curcuma Salisb.*, the rhizome of turmeric (*C. aromatica L.*), curcumin has antioxidation, anti-inflammation, antivirus, antitumor, and anticoagulation effects. Curcumin's therapeutic effect on uveitis is related to its antioxidant, anti-inflammatory, and antifibrinolysis properties. Kowluru and Kanwar [84] found that curcumin had antioxidant properties and could downregulate IL-1*β* and VEGF levels. Curcumin inhibited the release of IL-1, IL-6, IL-8, and tumor cytokine-*α* (TNF-*α*) by inhibiting NF-*κ*B expression, thus protecting iris ciliary cells and retinal pigment epithelial cells from inflammatory responses induced by lipopolysaccharide [85]. Curcumin inhibited choroid and retinal neovascularization by inhibiting vascular endothelial growth factor receptor [86]. Zhang et al. found that curcumin eye drops (10 mg/mL) administrated for 2 weeks could inhibit the axis of stromal cell-derived factor-1 (DF-1) and CXC chemokine receptor 4 (CXCR-4), protect retinal ganglion cells, significantly improve vitreous turbation, and inhibit retinal detachment [87]. Also, curcumin not only inhibited the proliferation of retinal pigment epithelial cells and epithelial-mesenchymal transition by inhibiting AKT, MAPK, and TGF-*β* pathways [88] but also protected retinal pigment epithelial cells from oxidative stress damage by upregulating heme oxygenase-1 (HO-1) and reducing ROS levels [89]. Curcumin's antioxidation effect is related to regulating Nrf-2/HO-1pathways.

Retinal ischemia-reperfusion injury (RIRI) is common in patients with uveitis and can cause retinal structure and function disorders. By regulating the Bcl-2/Bax/caspase-3 signaling pathway, curcumin could downregulate the expression of Bax and caspase-3 proteins and upregulate the expression of Bcl-2 proteins [90, 91]. Besides, curcumin could downregulate the expression levels of IL-23 and IL-17 in the retina.

In terms of clinical studies, Lal et al. [92] treated 18 patients with chronic anterior uveitis with curcumin (375 mg TID). After 12 weeks of continuous treatment, vision got improved in all patients, and pain, redness of eyes, congestion of the ciliary body, keratinized deposits, aqueous humor, and vitreous opacity disappeared without adverse reactions. Allegri et al. [93] included 106 patients with recurrent uveitis after receiving glucocorticoids, immunosuppressants, antiherpetic drugs, and nonsteroidal anti-inflammatory drugs (NSAIDS) before participated in this study. They had the patients take curcumin phosphatidylcholine complex (Meriva, 1200 mg, bid) orally, in addition to the former medications. After 12 months, only 19 patients relapsed. The study also found that oral administration of curcumin phosphatidylcholine complex significantly improved symptoms such as eye pain, blurred vision, pericorneal congestion.

In recent years, to solve the problem of curcumin's poor solubility and bioavailability, a variety of different dosage forms were reported besides curcumin phosphatidylcholine compounds, including curcumin/sodium alginate hydrogel nano-emulsion [94], curcumin nano-emulsion [95], new curcumin chitosan nanoparticle capsules [96], curcumin liposomes [97], and curcumin nanoparticles [98]. Dosage forms above were proved to improve the absorption of curcumin.

## 4. Summary

Natural products play a therapeutic role in uveitis in various ways, including anti-inflammation, antiapoptosis, antioxidation effects, and inhibiting neovascularization. Pharmacological actions on uveitis and related signal pathways of natural products have been summarized in Table 3. Anti-inflammatory effects are the main ways how natural products treat uveitis. Regulating TLR4/NF-*κ*B pathway can protect uvea from the destruction of inflammatory cells and cytokines induced by LPS. Plant polysaccharides, like APS and HPS, have structures similar to lipopolysaccharide. They can inhibit the production of downstream inflammatory factors caused by overexpression of the TLR4/NF-*κ*B signaling pathway in vivo. Besides, curcumin and matrine can also protect uvea from the damage of inflammatory reaction by regulating the TLR4/NF-*κ*B pathway. Th17 cells are the main pathogenic cells that mediate autoimmune diseases, including psoriasis, uveitis, and rheumatoid arthritis. Berberine can improve Behcet's disease symptoms by regulating the IL-17 signaling pathway. Tumor necrosis factor-related receptor 6 (TRAF6) is a kind of adaptor protein, which can conduct signals mediated by many receptors on the membrane, including the Toll/IL receptor family. LPS stimulation can induce the ubiquitination of TRAF6 and then form a complex with transforming growth factor-beta activated kinase 1 (TAK1), which further activates the IKKS family and promotes the nuclear transfer of NF-*κ*B, leading to the occurrence of inflammatory reactions. APS and HPS can protect retinal ganglion cells from damage of inflammatory response by regulating the TRAF6/TAK1 signaling pathway. IL-37 has a significant positive correlation with disease activity of HLA-B27 associated acute anterior uveitis (AAU). TWP can upregulate anti-inflammatory cytokine IL-37 expression by regulating ERK/MAPK signaling pathway. Moreover, the regulatory effects of natural products on cytokines have been summarized in Table 4.

In terms of antiapoptosis, Fas/FasL expression on T cells' surface is associated with uveitis. FasL expressed in the corneal epithelium blocks inflammatory cells from the conjunctiva and anterior chamber; FasL expressed in the iris ciliary body blocks inflammatory cells from invading blood vessels; FasL expressed in the retina induces rapid apoptosis of invading inflammatory cells, which is important for the protection of visual function and also has a killing effect on invading lymphocytes. By regulating Fas/FasL signaling pathway, TGP can inhibit the apoptosis of retinal cells. Caspase-3 is one of the important apoptotic executors in the caspase family. It is activated by protein hydrolysis in response to various apoptotic signals and promotes apoptosis in ocular tissue cells. By regulating the Bcl-2/Bax/caspase-3 signaling pathway, curcumin can inhibit the retinal cell apoptosis induced by endoplasmic reticulum stress and inflammatory cell invasion.

Oxidative stress can cause mitochondrial DNA damage, protein nitrification, and membrane lipid oxidation in uveal tissue. Heme oxygenase-1 (HO-1) is a ubiquitous and redox-sensitive induced stress protein that can protect cells from oxidative stress. With strong antioxidant activity similar to vitamins C and E, curcumin can protect RPE cells from oxidative stress by regulating the Nrf-2/HO-1 signaling pathway.

Ocular neovascularization is one of the main causes of severe visual impairment. Regulated by a series of molecular signals, neovascularization is a complex multistep process in which endothelial cells of mature vessels proliferate, migrate, and gradually remodel to form new small vessels. Curcumin and matrine inhibit choroidal, retinal, and iris neovascularization by regulating VEGF signaling pathway. The interaction between SDF-1 secreted by corneal stromal cells, epithelial cells, and inflammatory cells and its receptor CXCR4 are involved in regulating corneal wound repair and inflammatory corneal neovascularization proliferation. Curcumin can protect retinal ganglion cells by inhibiting the SDF-1/CXCR4 signaling pathway.

Although the total glucosides of paeony, TWP, and curcumin have their evidence in clinical trials, the available literature has a high risk of research bias. Natural products like berberine, matrine, and Astragalus polysaccharide are only reported in animal experiments in the field of uveitis. Randomized controlled clinical trials of the natural products above are still lacking. Their efficacy and safety in children and elderly patients remain uncertain.

## 5. Conclusions

Natural products have been proved effective in the treatment of uveitis in animal experiments. Further efforts are still needed to explore the therapeutic effects of natural products in clinical practice and search for new drugs with anti-inflammatory effects.

## Figures and Tables

**Table 1:** Search strategy in PubMed.

Number	Search terms
1	Uveitis (MeSH Terms)
2	Uveitis (ALL field)
3	Panuveitis (MeSH Terms)
4	Panuveitis (ALL field)
5	1 OR 2 OR 3 OR 4
6	natural product [MeSH Terms]
7	natural product [ALL field]
8	Matrine [ALL field]
9	Berberine (MeSH Terms)
10	Total glucosides of paeony [ALL field]
11	Tripterygium wilfordii polyglycoside [ALL field]
12	Astragalus polysaccharide [ALL field]
13	Hedysari polysaccharide [ALL field]
14	Rhubarb polysaccharide [ALL field]
15	Curcumin [ALL field]
16	6 OR 7 OR 8 OR 9 OR 10 OR 11 OR 12 OR 13OR 14 OR 15
17	5 AND 16

**Table 2:** Search strategy in EMBASE.

Number	Search terms
1	Uveitis (ti,ab,kw)
2	Panuveitis (ti,ab,kw)
3	Uveitis
4	Panuveitis
5	1 OR 2 OR 3 OR 4
6	natural product [ti,ab,kw]
7	natural product
8	Matrine [ti,ab,kw]
9	Berberine [ti,ab,kw]
10	Total glucosides of paeony [ti,ab,kw]
11	Tripterygium wilfordii polyglycoside
12	Astragalus polysaccharide [ti,ab,kw]
13	Hedysari polysaccharide [ti,ab,kw]
14	Rhubarb polysaccharide [ti,ab,kw]
15	Curcumin [ti,ab,kw]
16	6 OR 7 OR 8 OR 9 OR 10 OR 11 OR 12 OR 13OR 14 OR 15
17	5 AND 16

**Table 3:** Pharmacological actions on uveitis and related signal pathways of natural products.

Natural products	Pharmacological actions related to uveitis	Signal pathways	Symptoms that can be improved
Matrine	Anti-inflammation, inhibited proliferation of fibroblasts and neovascularization	TLR4-NF-*κ*B signaling pathway VEGF signaling pathway	Ciliary congestion, retinal edema, fundus hemorrhage, glaucoma, corneal neovascularization
Berberine	Anti-inflammation, regulation of gene expression, regulation of intestinal flora	IL-17 signaling pathway	Retinal edema and hemorrhage, corneal edema, retinal folding, iris congestion, iris adhesions
TGP	Anti-inflammation, antiapoptotic	Fas/FasL signaling pathway	Iris ciliary swelling, retinal edema, iris hemorrhage, hypopyon, miosis
TWP	Anti-inflammation, antiapoptotic	TLR-NF-*κ*B signaling pathway ERK/MAPK signaling pathway	Keratic precipitate, anterior chamber flare, iris edema, vision loss
APS	Anti-inflammation, antiapoptotic	TLR-NF-*κ*B signaling pathway TRAF6/TAK1 signaling pathway	Glaucoma
HPS	Anti-inflammation	TLR-NF-*κ*B signaling pathway TRAF6/TAK1 signaling pathway	Hypopyon, vision loss
RP	Anti-inflammation	TLR-NF-*κ*B signaling pathway	Unknown
Curcumin	Antioxidation, anti-inflammation, antiapoptotic, antifibrinolytic effects	TLR4-MAPK/NF-*κ*B signaling pathway SDF-1/CXCR-4 signaling pathway Bcl-2/Bax/caspase-3 signaling pathway Nrf-2/HO-1 signaling pathway	Keratic precipitate, eye pain, blurred vision, ciliary congestion, aqueous and vitreous opacity

**Table 4:** Regulatory effect of natural products on cytokines in uveitis.

Cytokines	Major secretory cells	Effect of cytokines in uveitis	Regulatory effect of natural product components
IL-1	Macrophages, epithelial cells	IL-1 can promote the activation of CD4 + T cells and the expression of IL-2 receptor 2 and the antigen presentation ability of APC such as monocyte macrophage [10]. Synergized with IL-2 or interferon, IL-1 can enhance NK cell activity. Moreover, it can recruit neutrophils and promote the release of inflammatory mediators [99]. Intravitreal injection of recombinant IL-1 receptor antagonist anakina (ANA) can inhibit the increase of laser-induced neovascularization choroidal area in a concentration-dependent manner and improve the uveitis symptoms such as iris edema, adhesion, atrophy, and neovascularization [100].	Matrine ↓ Berberine ↓ TWP ↓ APS ↓ HPS ↓ RP ↓ Curcumin ↓
IL-2	T cells	Il-2 can stimulate the proliferation and differentiation of Th17 cells, activate NK cells, and macrophages [101].	
IL-4	T cells, mast cells	IL-4 can induce the initial T cells to differentiate into Th2 cells and participate in the humoral immune response. Moreover, it can promote the proliferation and differentiation of activated B cells and induce immunoglobulin E (IgE) antibodies' production [102, 103].	TGP ↑
IL-6	T cells, macrophages, endothelial cells	IL-6 mediates the differentiation of Th1 to Th17 cells and inhibits physiological intraocular T cell apoptosis [104]. Intravitreal injection of anti-IL-6 (MP5-20F3) twice significantly relieved experimental autoimmune uveitis in mice [105].	Matrine ↓ Berberine ↓ TWP ↓ HPS ↓ Curcumin ↓
IL-8	Monocytes, macrophages, endothelial cells, fibroblasts, T cells	IL-8 takes part in chemotactic signals to recruit leukocytes, leading to directional migration and exocytosis of stored proteins [106, 107]. Intravitreal injection of IL-8 (100 ng) can induce uveitis in the rabbit [108]. Anti-IL-8 antibody treatment partially treated EIU in rabbits [109]. Gene polymorphisms of IL-8 may lead to different susceptibility to ocular Behcet's disease OBD and increase the risk of developing the disease [110]. IL-8 was found to be the best marker for the diagnosis of children's idiopathic anterior uveitis [111].	Berberine ↓ Curcumin ↓
IL-10	Monocytes	IL-10 can inhibit the expression of major histocompatibility complex (MHC) and costimulatory molecules in APC and inhibit the production of cytokines by activated Th1 cells [76]. IL-10 polymorphisms +434 T/C, +504G/T, and -2849C/T are predisposing factors for uveitis in children [112].	TWP ↓ HPS ↑ RP ↓
IL-12	Macrophages, dendritic cells	IL-12 can stimulate T cells and NK cells to produce IFN-*γ* and promote CD4 + helper T cells to differentiate into Th1 cells that produce IFN-*γ* [113].	
IL-17	Th17 cells, NK cells, CD8 T cells, neutrophils	As a proinflammatory cytokine, IL-17 can recruit and activate neutrophils and has synergistic effects with TNF, IL-1*β*, IFN-*γ*, granulocyte-macrophage colony stimulating factor (GM-CSF), and IL-22 [114, 115].	Berberine ↓ RP ↓ Curcumin ↓
IL-18	Activated macrophages	Interleukin-12 and interleukin-18 synergically promote the production of interleukin-17A and interleukin-17F, which is independent on IL-23 [116]. IL-18 was found to be a good biomarker for monitoring activity and regression of uveitis [117].	
IL-21	Th2 cells	IL-21 promotes the differentiation of Th17 cells that participate in the pathogenesis of autoimmune diseases such as scleritis, uveitis, and Behcet's disease [118]. Also, it can promote the proliferation and differentiation of B cells, NK cells, and effector CD8 + T cells [119, 120].	Berberine ↓ TWP ↓
IL-23	Macrophages, dendritic cells	IL-23 participates in the occurrence, recurrence, and chronicity of uveitis by promoting the production of IL-17. Moreover, it takes part in the recruitment and differentiation of myeloid cells, which is considered an upstream pathway in intermediate uveitis pathogenesis [121].	Berberine ↓ TWP ↓ Curcumin ↓
IL-27	Macrophages, dendritic cells, monocytes	IL-27 promotes the differentiation of Th1 but inhibits the proliferation of Th2, Th17, and Treg cells [109].	
IL-32	NK cells, macrophages, monocytes, and T lymphocytes, epithelial cells, endothelial cells, mesenchymal stromal cells, fibroblasts, and hepatocytes	IL-32 can induce proinflammatory cytokines like TNF-*α*, IL-8, and IL-1*β* and induce anti-inflammatory cytokines like IL-10 [122]. Moreover, it can mediate the differentiation of monocytes into dendritic cells [123].	
IL-33	Endothelial cells, smooth muscle cells	Both IL-33 and IL-33R were expressed in RPE cells, IL-33 can inhibit the production of IFN-*γ*, and IL-17 promote Th2 to secrete cytokines and significantly reduce the severity of EAU mice [124, 125].	
IL-35	Regulatory T cells	IL-35 can significantly increase the expression of IL-10 and TGF-*β* and decrease the expression of INF-*γ*, IL-12, and IL-17 [126]. Moreover, it can promote Treg cells' proliferation and inhibit the proliferation of Th17 cells [127].	
IL-37	Epithelial cells, dendritic cells, monocytes	IL-37 significantly inhibits IL-1*β*, IL-6, IL-10, IL-21, IL-23, TNF-*α*, and IFN-*γ*. IL-37 has a significant positive correlation with disease activity of HLA-B27 associated acute anterior uveitis (AAU) [61] as well as chronic primary angle-closure glaucoma [128].	TWP ↓
TNF-*α*	Macrophages, T cells, NK cells	TNF-*α* can directly kill cells infected by virus, activate monocyte macrophages, and enhance their phagocytic and bactericidal ability. Moreover, TNF-*α* can promote antigen processing and presentation pathways and increase Th1 and Th17 cytokines level [129]. Adalimumab and infliximab have become the most widely used biological agents in the treatment of noninfectious uveitis [130]. TGF-*β* can induce the differentiation of Th0 towards Treg and inhibit the differentiation of Th17 cells at high concentrations. At low concentrations, with the presence of IL-6, it can induce Th0 to differentiate into Th17 [131]. In patients with uveitis, the expression of TGF-*β* in aqueous humor decreases, which is considered a potential factor to promote uveitis. Similar changes are observed in the aqueous humor of patients with Vogt Koyanagi Harada during the active phase [132]. Adalimumab and infliximab have become the most widely used biological agents in the treatment of non- infectious uveitis [133].	Matrine ↓ TWP ↓ APS ↓ HPS ↓ RP ↓ Curcumin ↓
TGF-*β*	Monocytes, T cells, chondrocytes	TGF-*β* can induce the differentiation of Th0 towards Treg and inhibit the differentiation of Th17 cells at high concentrations. At low concentrations, with the presence of IL-6, it can induce Th0 differentiate into Th17 [131]. In patients with uveitis, the expression of TGF-*β* in aqueous humor decreases, which is considered a potential factor in promoting the development of uveitis. Similar changes are observed in the aqueous humor of patients with Vogt Koyanagi Harada during the active phase [132].	Berberine ↓
IFN-*γ*	T cells, NK cells	It can activate macrophages, promote MHC expression and antigen presentation, promote Th1 differentiation, and inhibit Th2 differentiation [133]. IFN-*γ* can induce VEGF expression in retinal cells through PI-3 K/Akt/mTOR/p70S6 kinase pathway [134]. Deficiency in IFN-gamma can inhibit the development of uveitis induced by muramyl dipeptide [133].	TGP ↓ TWP ↓ RP ↓
VEGF	Tumor cells	VEGF can not only promote the increase of vascular permeability and the degeneration of the extracellular matrix but also promote the neovascularization of choroid, iris, and retina, leading to severe visual loss, even blindness.	Matrine ↓ Curcumin ↓
MCP-1	Immature DC cells, monocytes/macrophages, T cells, NK cells	MCP-1 can recruit immature DC cells, T cells, and monocytes/macrophages to participate in immune response and inflammatory response. Alteration of MCP-1 in aqueous humor was associated with glaucoma secondary to Fuchs uveitis syndrome [8, 135].	Berberine ↓ TWP ↓
MMP-9	Neutrophils, monocytes/macrophages	MMP-9 can remodel the dynamic balance of the extracellular matrix and promote the release of TGF-*β*1 and VEGF [136]. MMP-9 levels peak at the most severe uveitis stage and then return to baseline as the inflammation subsides [136].	TWP ↓

## Data Availability

The data used to support the findings of this study are included within the article.
